# Overcoming the Boundaries of Heart Warm Ischemia in Donation After Circulatory Death: The Padua Case

**DOI:** 10.1097/MAT.0000000000002141

**Published:** 2024-02-09

**Authors:** Gino Gerosa, Paolo Zanatta, Annalisa Angelini, Marny Fedrigo, Roberto Bianco, Demetrio Pittarello, Tea Lena, Alessia Pepe, Giuseppe Toscano, Fabio Zanella, Giuseppe Feltrin, Nicola Pradegan, Vincenzo Tarzia

**Affiliations:** From the * Cardiac Surgery Unit, Cardio-Thoraco-Vascular and Public Health Department, Padova University Hospital, Padova, Italy; † Department of Critical Care, Anesthesiology and Intensive Care Unit, Ca’ Foncello Hospital, Treviso, Italy; ‡ Cardiovascular Pathology, Cardio-Thoraco-Vascular and Public Health Department, Padova University Hospital, Padova, Italy; § Cardiac Surgery Anesthesiology and Intensive Care Unit, Cardio-Thoraco-Vascular and Public Health Department, Padova University Hospital, Padova, Italy; ¶ Institute of Radiology, Department of Medicine, University of Padua, Padua, Italy; ∥ Regional Health Department, Regional Transplant Center, Veneto Region, Italy.

**Keywords:** DCD, heart transplantation, organ conditioning

## Abstract

A 45 year old male obese patient with a previous history of repaired congenital heart disease developed worsening heart failure making heart transplantation listing mandatory. Unfortunately, due to his anthropometric measures, the search for a suitable brain-dead donor was unsuccessful. For this reason, he accepted to be enrolled in the controlled donation after circulatory death (cDCD) program. According to the Italian Law regulating death declaration after cardiac arrest (no-touch period of 20 minutes—one of the longest in the world), we faced a 34 minute cardiac asystole, after which the heart was recovered through a thoraco-abdominal normothermic regional perfusion excluding the epiaortic vessels. The heart was then preserved by means of cold static storage. Heart transplantation was performed successfully without any signs of primary graft failure. Postoperative endomyocardial biopsies were negative for acute cellular and antibody-mediated rejection. Furthermore, echocardiographic and cardiac magnetic resonance evaluation of the heart did not show any functional abnormalities. The patient was discharged on post-operative day (POD) #39 in good clinical conditions.

A 45 year old male patient with a known history of repaired congenital heart disease (CHD) was referred to our Advanced Heart Failure Program for worsening dyspnea despite optimal medical therapy. The patient had undergone surgical repair of a partial atrioventricular canal defect at the age of 1 year. During the regular cardiological follow-up, the patient developed severe right and left atrioventricular valve regurgitation, residual left-to-right interatrial shunt, and atrial arrhythmia (flutter). He underwent a redo surgery (right and left atrioventricular valve repair, residual interatrial defect repair with a patch, cardiac ablation) at the age of 39 years. The postoperative course was complicated by a complete heart block requiring pacemaker implantation. Despite optimal medical therapy, the patient during the follow-up developed worsening dyspnea (New York Heart Association [NYHA]—Function Class III) and reduced ejection fraction heart failure (HFrEF). Besides, a cardiopulmonary test showed a maximal oxygen consumption (VO_2_ max) = 9.8 ml/kg/min. After multidisciplinary discussion, the patient was listed for heart transplantation in October 2020. Unfortunately, given his anthropometric measurements (body mass index = 34 kg/m^2^), a brain-dead donor (DBD) with a proper donor-recipient match was never available. For this reason, the patient was the one with the longest time on the waitlist for his blood group up to May 2023.

The use of controlled donors after circulatory death (cDCD) for heart transplantation has been questioned for years in Italy given the longest in the world no-touch period after death declaration (20 minutes).^1^ However, in April 2023 the National Transplant Centre (CNT) approved a multidisciplinary document to guide heart donation in cDCD.^2^ On May 10, 2023 a 55 male cDCD donor was available for heart donation and, given his body measurements (weight = 100 kg, height = 185 cm), he was deemed eligible for our patient. After signing the informed consent, the patient underwent heart transplantation from a cDCD donor on May 11, 2023.

## Materials and Methods

### Donor Characteristics

The donor was a 55 year old male patient who suffered severe polytraumatism after a motorcycle accident 3 weeks before donation. Brain imaging at hospital admission showed diffuse cerebral edema, and despite neurosurgical drainage, there were no neurologic and radiological improvements during the following days after hospitalization. Despite his poor prognosis, he never met the clinical criteria for brain death declaration. However, given his prognosis and in light of the clear statement of the family members favorable to donation, the decision to withdraw life support (WLS) and pursue donation after circulatory death was finally taken.

Liver, kidneys, and heart were considered suitable for donation, whereas lungs were deemed ineligible. A transthoracic echocardiogram showed a preserved biventricular function, no valve regurgitation, no ventricular dilation or hypertrophy. A coronary angiogram did not show any significant coronary lesions. The donor was hemodynamically stable without any inotropic support at the time of WLS.

### Surgical Strategy for Heart Recovery, Explant, and Implant

All the technical maneuvers were performed in respect of the CNT multidisciplinary document.^2^ Before WLS initiation, the patient was transferred to the operating room (O.R.) (to minimize the ischemia time), intubated, and with one femoral vein and two femoral artery 10fr vascular sheaths in place. The donor was then prepared and heparin was administered. After the 20 minute no-touch period, the retrieval surgical group decided to establish a thoraco-abdominal normothermic regional perfusion (TA-NRP). For achieving this, we used a conventional cardiopulmonary bypass (CPB) pump. Conventional cardiopulmonary bypass included one femoral vein cannula, one femoral artery cannula, one cardioplegia line, one cardiac vent line. Indeed, a CytoSorb filter was mounted on the pump. Conventional cardiopulmonary bypass priming included albumin, mannitol, and red blood cells. Thoraco-abdominal normothermic regional perfusion included an initial phase after the no-touch period during which the femoral vessels were cannulated, CPB was instituted and an endoaortic occluding balloon was inserted in the thoracic aorta, while the sternum was opened. Once exposed, the ascending aorta was cross-clamped and perfused through a cardioplegic cannula using type C Buckberg solution, and a right pulmonary vein vent was inserted to unload the left chambers. In the meanwhile, the epiaortic vessels were identified and clamped, followed by deflation of the endoaortic balloon. Once the C solution was completely administered, the aortic cross-clamp was released. After an initial period of reperfusion (during which the unloaded heart regained a sinus rhythm), the donor was weaned from CPB. Cardiac functionality was established according to hemodynamic and electrocardiogram (EKG) criteria (mean arterial pressure >50 mm Hg, and no arrhythmias, respectively) and echocardiographic parameters (ejection fraction >50%, tricuspid annular plane excursion (TAPSE) >20 mm, no valve abnormalities, no regional alterations of left ventricular wall motion). Once deemed eligible for donation, the heart was stopped conventionally with cold Celsior cardioplegia and preserved with cold storage.

Cardiectomy was performed in the usual manner, and the heart implantation was performed according to a bicaval technique.

## Results

### Donor Heart Retrieval Times

**Figure 1.**
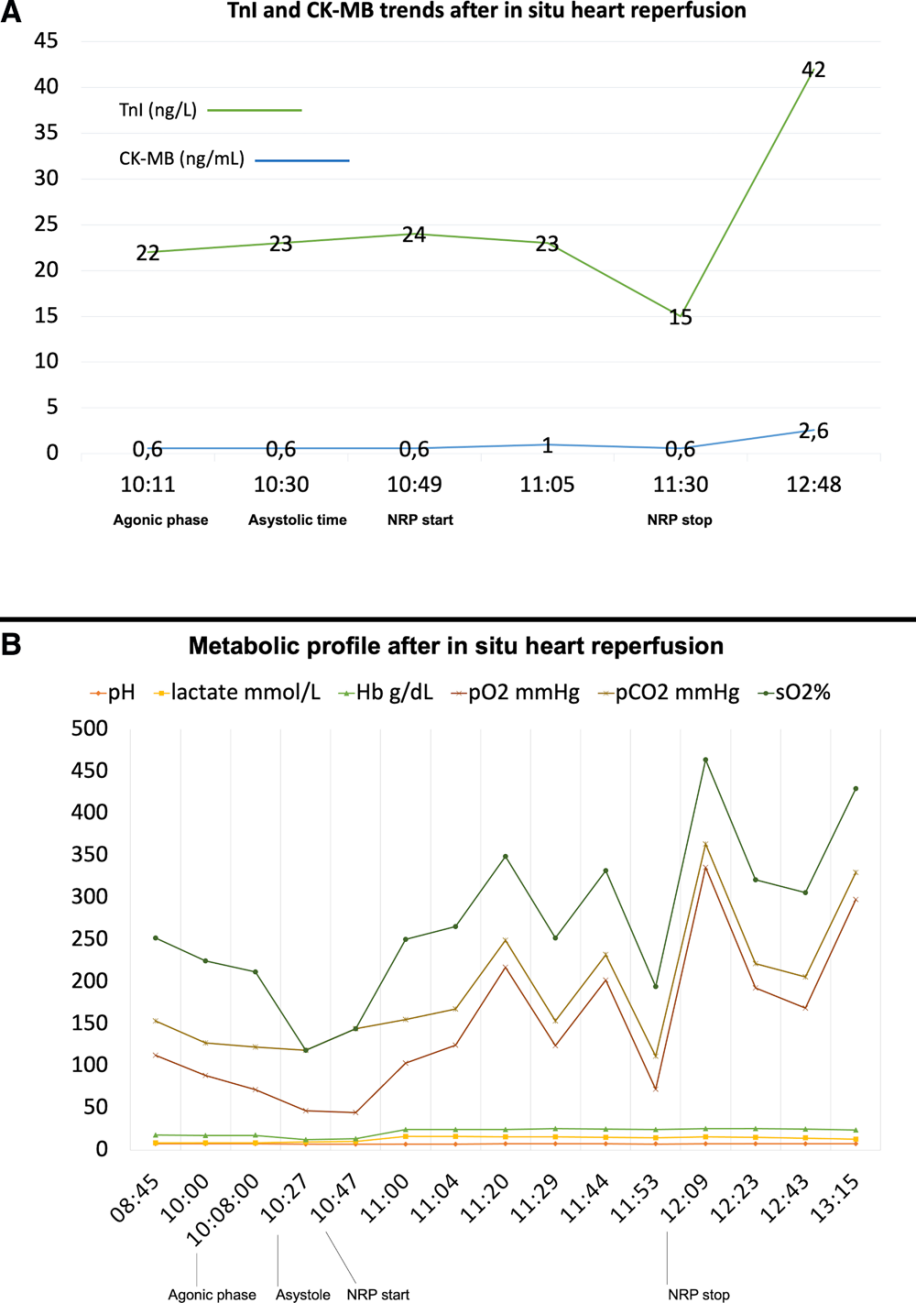
Laboratory assessment during heart donation after circulatory death. **A**: Trends of troponin and CK-MB levels soon after *in situ* reperfusion of the donor heart. **B**: Metabolic profile after *in situ* reperfusion of the donor heart. CK-MB, creatine kinase-myoglobin binding.

The agonic time was 19 minutes; the functional warm ischemia time (fWIT) was 47 minutes; the asystolic period (declaration of death to reperfusion) was 34 minutes. After 69 minutes of reperfusion, CPB was weaned and the heart was evaluated for further 108 minutes. During this phase, the donor was maintained on low dose of inotropes (IV norepinephrine 0.05 mcg/kg/min) maintaining a normal blood pressure (110/50 mm Hg) and stable sinus rhythm (70 beats/min); a transesophageal echocardiogram showed preserved biventricular function, no valve regurgitation, and mild interventricular septal hypertrophy (secondary to edema) (Video 1, Supplemental Digital Content). The donor metabolic profile is synthesized in Figure 1. The cold ischemia time after Celsior solution was 132 minutes.

**Video 1.** 

### Early Postoperative Course

After aortic clamp removal, the implanted heart regained a normal sinus rhythm; after a 60 minute period of warm reperfusion, the recipient was easily weaned from CPB.

The patient was transferred to intensive care unit (ICU) with a low dose of inotropes (IV epinephrine and norepinephrine 0.05 mcg/kg/min) in stable conditions. The patient did not experience any primary graft failure in the first 48 hours after surgery. He was extubated on post-operative day (POD) #1, but he required reintubation for acute respiratory failure. He was definitively extubated on POD #7, and was transferred to the floor on POD #13. The patient was discharged home in good clinical conditions on POD #39.

Immunosuppressive therapy included thymoglobulines + steroids for 3 days, followed by cyclosporin + steroids.

**Figure 2.**
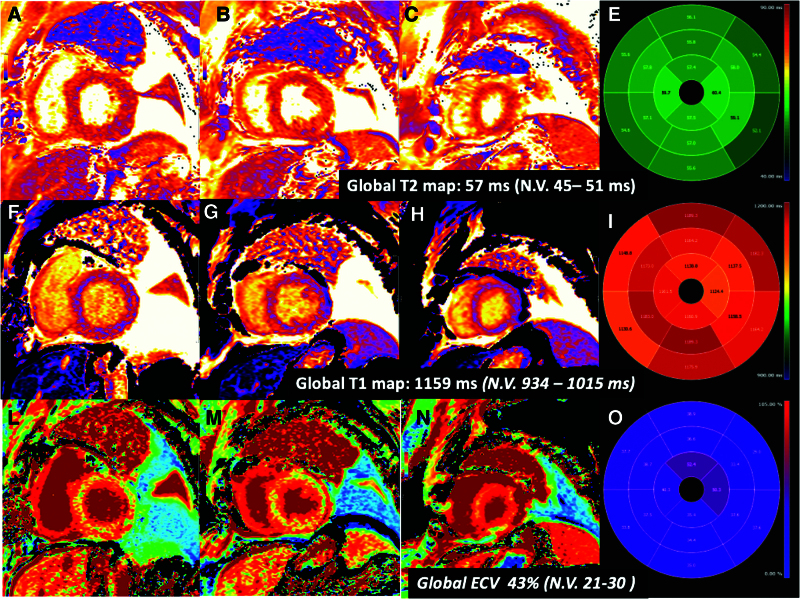
Posttransplant cardiac magnetic resonance. Basal (**A, F, L**), mid (**B, G, M**), and distal (**C, H, N**) short axis slice T2 mapping (**A–C**), T1 mapping (**F–H**), ECV (**L–N**). Bull’s-eye representation of the native T2 values (**E**), T1 values (**I**), and ECV (**O**) in the 16 myocardial segments (according to the American Heart Association). ECV, extracellular volume values.

**Figure 3.**
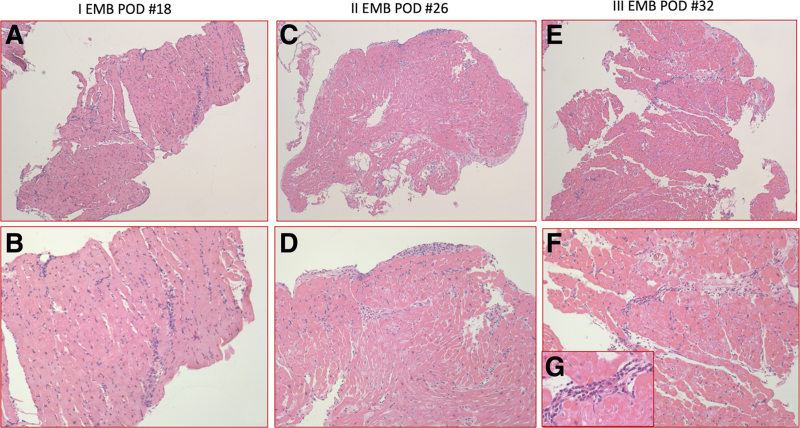
Posttransplant endomyocardial biopsies. Microscopic view of postoperative endomyocardial biopsies. **A, B**: First EMB (one of the three fragments) showing mild interstitial inflammatory lymphomonocytic infiltration not associated to necrosis and mild ischemic damage, with focal cardiomyocytes damage. C4d and CD68 were negative for AMR. **A**: Hematoxylin–Eosin stain, original magnification ×5. **B**: High power view showing the nature of inflammatory cells infiltration, ×10. **C, D**: Second EMB (one of the three fragments) showing absence of acute cellular rejection, mild ischemic damage with early reparative changes in the subendocardium. **C**: Hematoxylin–Eosin stain, original magnification ×5. **D**: High power view of **C**, original magnification ×10. **E–G**: Third EMB (one of the three fragments) showing again mild focal ischemic damage with packed granulocytes in the vessels and in the perivascular space. **E**: Hematoxylin–Eosin stain, original magnification ×5. **F**: High power view of **E**, original magnification ×10. **G**: Inset highlighting the granulocytes. AMR, acute myocardiac rejection; EMB, endomyocardial biopsies.

Cardiac magnetic resonance (CMR) on POD #20 documented ventricles with normal volumes, preserved regional and global systolic function, strain indexes, and wall thickness, no myopericardial late gadolinium enhancement (Figure 2). Endomyocardial biopsies performed on POD #18, POD #26, and POD #32 found grade 1R, grade 0, and grade 0 cellular rejection, respectively (according to current International Society of Heart and Lung Transplantation guidelines^3^). Focal signs of myocardial ischemic damage were found (Figure 3).

At 4 months of follow-up, the patient is alive, in good clinical conditions (NYHA Class I), in sinus rhythm, without any cellular or humoral rejection signs or any other microscopic abnormalities at the last endomyocardial biopsy at the same follow-up time.

## Discussion

Even if heart procurement in cDCD has been reported for longer fWIT (>30 min) with early clinical outcomes similar to DBD or cDCD with short fWIT,^3,4^ this is the first case in the world reporting such an extremely long controlled cardiac asystolia before using the heart for transplantation. Within the fWIT, the asystolic period is the time included between the circulatory death declaration (no-touch period onset) and the cardiac reperfusion initiation. During this period, the heart does not have both electrical and mechanical activity and it is not perfused. The asystolic period is primarily determined by the no-touch period which is established by each country based on local regulations. Our Nation presents the longest no-touch period in the western world (20 minutes)^1^ because this time is considered mandatory for the concept of death as irreversible cessation of brain function. In the meanwhile, it might be a threat for organ preservation in the context of cDCD, in particular for the heart which is profoundly susceptible to time-dependent warm ischemia injury.^5,6^

Longer periods of asystolia have been typically considered prohibitive for adequate heart function recovery,^7^ but our case has crossed the boundaries of almost 30 minute asystolia which was considered a contraindication for the use of the heart.

According to our results, the observed satisfactory recovery of the cardiac function is probably due to the specific strategy of WLS and a pharmacological preconditioning protocol given at different times during the DCD process (Table 1). In particular, the drugs used in our protocol address the main concerns of cDCD: postcirculatory arrest vasoplegia and organ ischemic-reperfusion injury. Regarding the sedation protocol, the hypoxic sympathetic storm was anticipated and treated by a specific sedation-anesthesia ward protocol based on remifentanil/propofol target controlled infusion (TCI) modality which is in accordance with end-of-life ethical principles and palliative sedation Italian guidelines.^8^ Regarding the use of a different extracorporeal circuit, further data are required to understand if ExtraCorporeal Membrane Oxygenation (ECMO) gives similar results to CPB in DCD scenario for heart transplant.

**Table 1.** Drugs Regimen Used During Controlled Donation After Circulatory Death

Preconditioning Protocol		
Days Prior Withdrawal	Withdrawal of Life Support	After Declaration of Death
Continuous IV infusion fentanil 1 µg/kg/h	Continuous infusion IV fentanil 1–2 µg/kg/h	Continuous infusion fentanil 1–2 µg/kg/h
80 mg dosage of atorvastatina	Methylprednisolone IV bolus 15 mg/kg	NAC 5 gm
10 mg melatonine	NAC 25 gm	CPB priming implemented with 6 fl albumine + 2 packed red cells + bicarbonates 200 mEq + CytoSorb filter
Vitamin C 1 gm bid	Heparin 300 UI/kg	
NAC 600 mg tid	TCI sedation with propofol	
	TCI sedation with remifentanil (dosage 0–2 mg/kg) During the asystolic time infusion rate reduction of 70%	Low fraction of oxygen Low calcemia not compensate
	If heart available for transplantation sevoflurane (MAC 0.5) until the stop of ventilation	If heart available for transplantation sevoflurane (MAC 0.5) until explant

CPB, cardiopulmonary bypass; MAC, minimum alveolar concentration; NAC, N-acetyl-L-cysteine; TCI, target controlled infusion.

The postoperative morpho-functional assessment of the transplanted cDCD heart by means of echocardiogram, CMR, biopsies, excluded any functional and macroscopic damages.

To the best of our knowledge, this is the first case in which a normal heart underwent controlled asystole for more than 30 minutes and was then recovered and successfully used for heart transplantation. An immediate heart recovery was possible after prompt reperfusion following the 20 minute no-touch period. This achievement was due to several reasons: use of medications with proved efficacy on cDCD ischemic-related damage; use of extracorporeal technologies able to recondition and recover ischemic grafts; application of ethical principles both for the donor and the recipient. This case might help in expanding the donor pool exceeding the current boundaries, whereas setting an important milestone for ischemic organs reconditioning.
